# Handwriting speed in juvenile idiopathic arthritis using the detailed assessment of speed of handwriting

**DOI:** 10.1186/s12969-024-01013-y

**Published:** 2024-08-15

**Authors:** C. A. Marchak, S. James, I. Davidson, J. Brown, K. Houghton

**Affiliations:** 1 Pediatric Resident Doctor, University of British Columbia, Vancouver, Canada; 2 Occupational Therapist, BC Children’s Hospital, Vancouver, Canada; 3 Physiotherapist, BC Children’s Hospital, Vancouver, Canada; 4 Pediatric Rheumatologist BC Children’s Hospital, Vancouver, Canada

**Keywords:** Handwriting, Speed, Juvenile idiopathic arthritis, DASH

## Abstract

**Background:**

Handwriting is a commonly reported functional limitation for children with juvenile idiopathic arthritis (JIA). The aim of this study was to evaluate handwriting in children with JIA.

**Findings:**

Twelve children (mean age 13.0 years, SD = 1.9; range 9.1 to 15.6 years) with JIA completed the Detailed Assessment of Speed of Handwriting (DASH). The presence of hand and wrist arthritis, grip strength, disability, pain, and quality of life (QOL) was also assessed. The mean DASH score was 34.5th percentile (*SD* = 22.5). Eight (75%) scored below the 50th centile. DASH scores were negatively associated with grip strength (*r* = -0.31).

**Conclusions:**

Handwriting difficulties are common in children with JIA. Handwriting assessment may be helpful to direct treatments, and advocate for support and accommodations in school.

**Supplementary Information:**

The online version contains supplementary material available at 10.1186/s12969-024-01013-y.

Hand and wrist involvement is common in juvenile idiopathic arthritis (JIA) and may cause difficulties in multiple areas of function. Previous studies have identified over half of children with JIA report hand and wrist problems at school, with handwriting the most reported functional limitation in daily activities [1, 2]. Assessment of handwriting is therefore important to identify children who will benefit from classroom supports and accommodations.

To better understand handwriting limitations in children with JIA, this pilot study aimed to (1) describe handwriting speed measured by the Detailed Assessment of Speed of Handwriting (DASH) [3], a standardized assessment of handwriting speed used by occupational therapists; (2) compare the DASH scores with age- and sex-matched normative data; and (3) explore associations between DASH scores and active wrist and hand arthritis, grip strength, quality of life, disability, and pain. We hypothesized that children with JIA will have a decrease in handwriting speed when compared to reported age-matched norms. Results will be used to inform larger scale studies and practice guidelines for handwriting assessments.

## Methods

As part of this study, children aged 9–17 diagnosed with JIA [4] and actively followed by the pediatric rheumatology clinic at BC Children’s Hospital underwent a standardized handwriting assessment by an occupational therapist (OT) using the Detailed Assessment of Speed of Handwriting (DASH) (Barnett et al., 2009) [3], and grip strength measurement by a physiotherapist (Citec handheld dynamometer). They also had a physician assessment of active joint counts (0–71 joints). Participant reported outcomes included questionnaires on function (Child Health Assessment Questionnaire, CHAQ), pain (visual analog scale [VAS]) and parent/patient global assessment of well-being (VAS). [5] Details of the study measures are summarized in Table 1. Children with a diagnosis of attention deficit hyperactive disorder, learning disability, or developmental coordination disorder were excluded.

**Table 1:** Study measures. DASH (detailed Assessment of Speed of Handwriting), CHAQ (Child Health Assessment Questionnaire)

DASH	Five tasks that mimic handwriting skills students perform at school including copying a sentence using best writing and fastest writing, writing the lower-case alphabet from memory, quickly and accurately drawing crosses within concentric circles, and free writing. The word per minute (WPM) score was calculated for each task and compared to normative data (30)
Grip Strength	Citec handheld dynamometer which was calibrated according to manufacturer specifications. Participants were seated with both feet on the ground. Their shoulder was adducted and held in line with the torso, the elbow was flexed to 90º with the wrist in neutral position. The participant was instructed to squeeze as hard as they can for 3 s. They repeated this a total of 3 times on each hand with 10 s of rest between repetitions. The best result of the three was used in analysis.
CHAQ	Participants completed the CHAQ as a self-reported measure of their functional impairment. The CHAQ describes disability attributed to the illness using three indicators (perceived difficulty, need for help from another person, or need for assistive devices), while performing activities of daily life (dressing and grooming, arising, eating, walking, hygiene, reach, grip, activities). As part of the CHAQ, they were asked to rate their pain in the past week on a 10-cm visual analogue scale (VAS) from 0 (no pain) to 10 (very severe pain) and global assessment of well-being, measured on a 10-cm VAS where 0 = very well and 10 = very poor (5).

**Table 2:** Patient characteristics. IEP (individualized education plan). SJIA (systemic juvenile idiopathic arthritis), ERA (enthesitis related arthritis), Poly RF- (polyarthritis rheumatoid factor negative), extended oligo (extended oligoarthritis), undiff (undifferentiated arthritis). All participants are right hand dominant except #5 who is left hand dominant

Participant	Age (y)	Sex	Grade	IEP	JIA subtype		Disease duration (y)	Hand or Wrist Arthritis		Medications		
1	13.5	F	8	Y	SJIA		5.1	History		Methotrexate, Infliximab		
2	13.8	M	8	N	ERA		2.8	No		Vimovo, Methotrexate, Infliximab		
3	11.2	M	6	N	ERA		1.2	No		Naproxen, Methotrexate		
4	9.1	F	3	N	Poly RF-		3.3	Active		Naproxen, wrist joint injection		
5	13.0	F	7	N	Extended Oligo		7.8	History		None		
6	14.3	F	8	N	ERA		2.1	No		None		
7	13.2	F	7	N	Undiff		11.5	No		Tocilizumab		
8	14.3	F	8	N	Psoriatic arthritis		5.7	History		Naproxen, Methotrexate, Adalimumab		
9	15.5	Trans M	9	N	Poly RF-		2.0	Active		Naproxen, Adalimumab		
10	15.4	M	9	N	ERA		3.7	History		Adalimumab		
11	10.3	F	5	N	Persistent Oligo		1.8	History		Methotrexate		
12	12.8	F	8	Y	ERA		2.5	Active		Celebrex, Methotrexate, Tofacitinib		

The University of British Columbia, Children’s and Women’s Research Ethics Board approved all procedures (certificate H21-00294). Parents or guardians provided written informed consent and children provided assent.

### Data Analysis

Descriptive statistics were used to describe the study population baseline characteristics and patient reported outcome measures including pain, quality of life and CHAQ score. A Pearson correlation was used to examine relationship between DASH score and grip strength.

### Findings

There were 12 participants, mean age 13.0 years, SD = 1.9; range 9.1 to 15.6 years; 8 girls, 3 boys, and one trans male youth. Three participants had active dominant hand/wrist arthritis and 5 participants had history of hand/wrist arthritis. No participants had radiographic damage or deformity. Table 2 summarizes descriptive characteristics of the cohort.

**Fig. 1:**
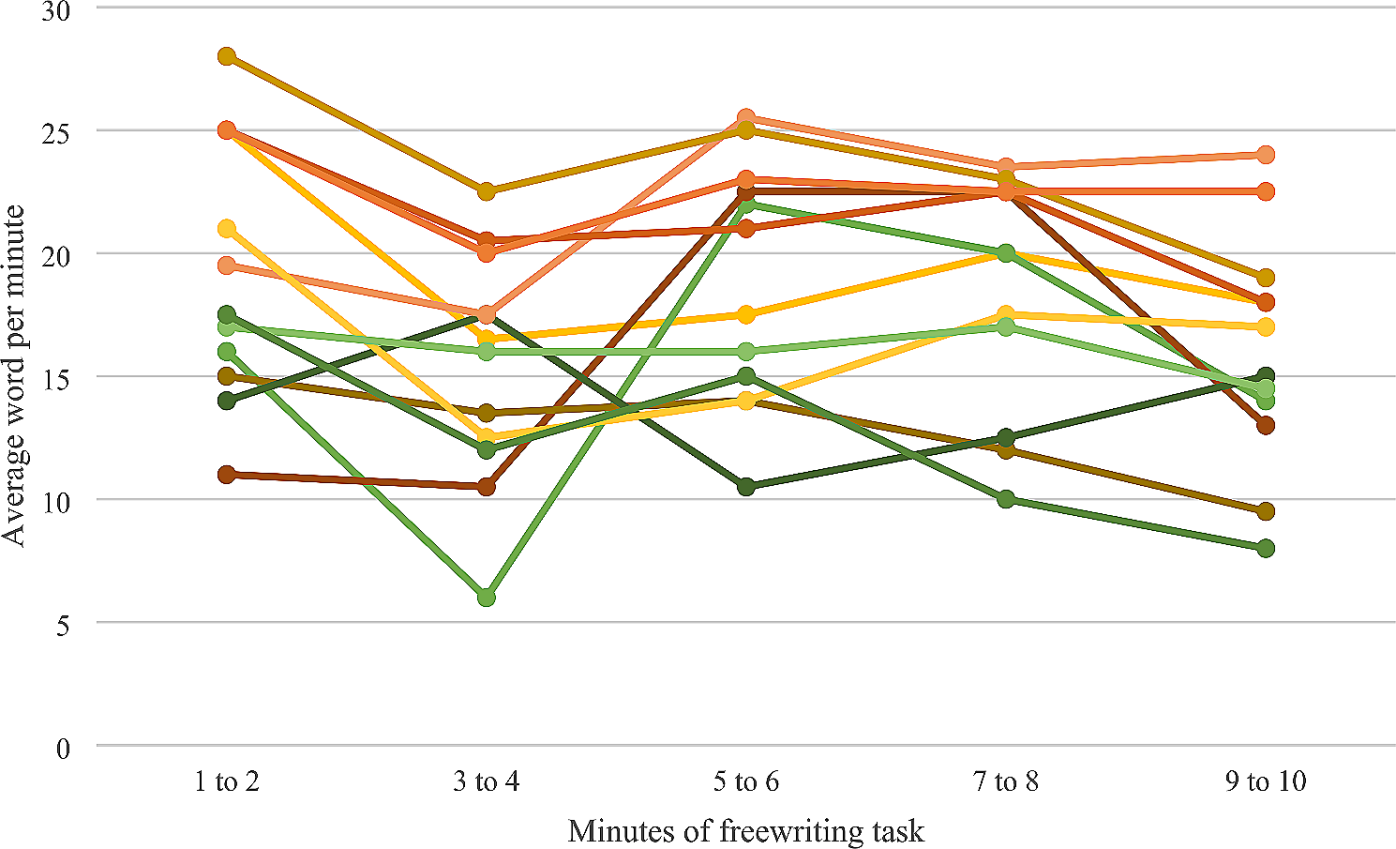
Summary of handwriting speed profiles of participants during 10-minute freewriting task

The mean DASH score of all participants was 34.53%ile (SD = 22.51, range 4.9 to 71.8). Eight (75%) scored below the 50th centile and 4 (33%) scored below the 16th %ile or 1 SD below the mean. DASH scores were negatively associated with grip strength (*r* = -0.31). During the extended handwriting task within the DASH, participants free write for ten minutes. There was an average speed of 17.53 words per minute (SD = 4.33wpm). The pattern of writing speed over the course of this task is illustrated in Figure 1.

The mean CHAQ score was 0.56 (SD 0.55, range 0 to 1.625). Five participants (42%) reported difficulty in the Grip domain question “Is your child able to write or scribble with pen or pencil?” with four reporting ‘with some difficulty and one reporting ‘with much difficulty’. The mean QOL score was 4.0 (SD 2.5, range 0 to 8.0) and mean pain score was 4.2 (SD 2.3, range 0 to 7.5). We compared participants who endorsed difficulty on the CHAQ grip domain question “Is your child able to write or scribble with pen or pencil?” to those who did not. The mean DASH score for those endorsing difficulty with writing on the CHAQ was 28.7 (SD 26.6) compared to 39.5 (SD 19.1) for those citing no difficulty with writing (p = 0.16, t-test).

## Discussion

In this study, we set out to measure handwriting speed in JIA using the Detailed Assessment of Speed of Handwriting (DASH). We have shown that handwriting difficulties are common and for multidisciplinary teams, OT and PT handwriting assessments are feasible to perform in the outpatient setting.

Handwriting is a complex activity requiring extensive neuromuscular coordination. Children with arthritis in their hands or wrist may have pain, weakness or limited range of motion. Children experiencing pain can become fatigued and distracted. They may change how they grip a pencil / pen to limit or avoid the pain of stiff joints. Hoeksma and colleagues reported a high prevalence of hand- and wrist-related symptoms and impairments in a cohort of 121 school aged children with JIA and low disease activity with 54% reporting hand- and/or wrist-related problems at school [6]. Pain was the most reported symptom and considerably more children reported symptoms and impairments than had physical exam findings of active arthritis or disease damage [6]. Haberfehlner and colleagues described a cohort of 15 children with JIA and self-selected handwriting difficulties performing a 5-minute handwriting test. The children reported pain during handwriting and had significant decrease in the numbers of letters they wrote per minute during the test. [1] The presence of arthritis, and limitation in grip force and wrist range of motion did not correlate with handwriting output. [1]

Consistent with the literature, we found that our cohort of children with JIA had impairments in handwriting with low mean DASH scores. Our numbers were too small to explore relationships between disease activity and handwriting. The CHAQ-DI grip domain question “Is your child able to write or scribble with pen or pencil?” did not capture all the participants who had DASH scores below the 50th percentile. This may be due to small participant numbers and insensitivity of the CHAQ-DI for handwriting activities. We noticed a decrease in writing speed over time during the DASH free writing 10-minute task. The scoring for this is divided into 2-minute sections and scored in words per minute for the whole 10-minute period. Thus, we are unable to comment on whether the pattern of handwriting speed in our participants with JIA differs from the normative population.

Handwriting is important for school participation and achievement. Children master letter formation at a relatively early stage in their school learning, with handwriting fluency steadily developing until attaining automaticity. The capacity theory of writing proposes that as automaticity develops, the effort dedicated to the mechanics of handwriting is reduced, freeing capacity for planning, composing and editing content. [7] A study of the handwriting ability of 284 primary school children aged 8 to 9 years old found correlations between poor handwriting, lower cognitive and literacy scores, and a longer duration for handwriting tasks [8]. Handwriting is not only important in school aged children; Summers and colleagues report impairments in handwriting speed in university students limits fluency of thought communication and exam completion. [9]

Children and youth with JIA may benefit from support and accommodation in the classroom. In 2019, Chomistek and colleagues performed a cross-sectional survey of 98 children with JIA aged 8 to 17, to identify patient-reported school barriers. They found 30% reported difficulties writing and 20% percent had accommodations at school including computer or tablet access, extra time for tests, or modified gym. [10]

**Fig. 2:**
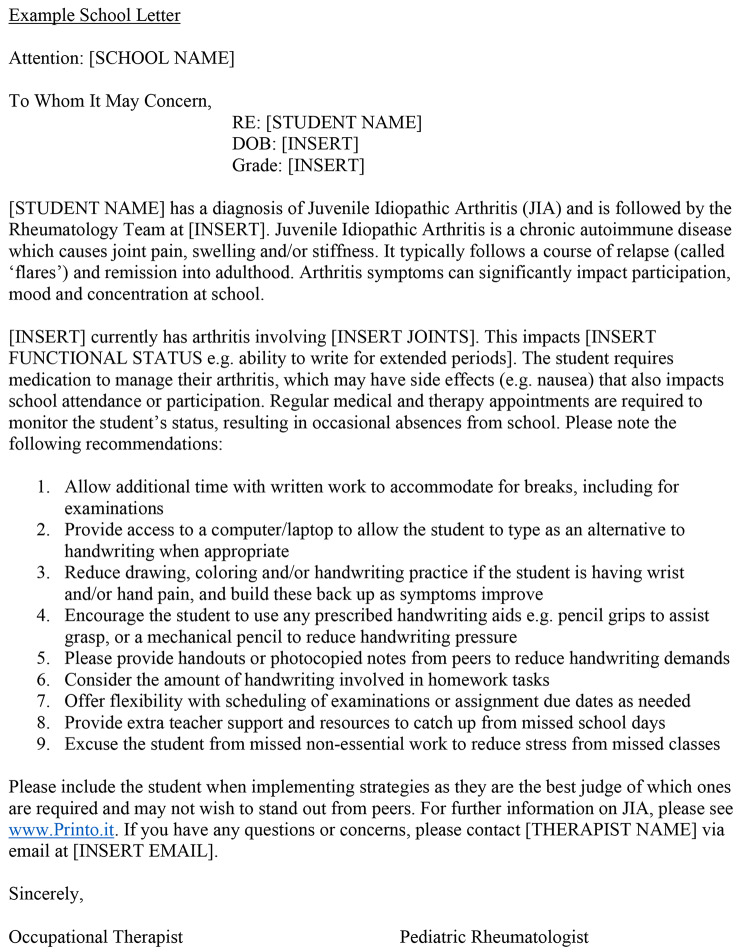
School letter example

A recent qualitative study in 2021, explored the experience of teachers supporting children aged 7 to 11 years old with JIA. They describe four key themes to best support children with JIA; communicating (with child, parents, healthcare team); flexing and adapting (ability to provide individualized support to maximize inclusion); inclusion (mindful child is not seen as ‘different’); and learning and knowing (continuing education). [11] These findings highlight the importance of a team approach to ensuring a child’s success in school with the child, parents, teacher, and healthcare professionals working together. It is our usual practice to provide school accommodation letters for all our patients with JIA with individual recommendations as per our OT, PT, and medical assessment. Figure 2 shows an example of a school letter, highlighting the need for pacing and additional time for written work.

## Study strengths and limitations

The strengths of our pilot study is the use of the DASH administered by our OT. The DASH is a valid and reliable measure that contains 5 tasks that mimic handwriting skills students perform at school. An OT has the skills required to administer the DASH, interpret the results, and provide a number of treatment options.

We acknowledge several limitations of our study. This is a pilot study with small numbers which limits our ability to examine associations between handwriting and other clinical variables. There is the potential for inclusion bias with children who have more difficulty with handwriting more likely to participate. We did not measure fatigue, or pain during handwriting, which may relate to handwriting impairments. Further studies with larger populations are needed to clarify the effects of arthritis disease activity, pain and fatigue on handwriting. Finally, we did not explore the effect of treatment, including accommodations in school, and this will be important to inform best practice.

## Conclusions

Handwriting speed difficulties are common in children with JIA. It is important to ask all children with JIA about handwriting, function, and school limitations at each clinical visit. If rheumatology teams have access to OT or PT, handwriting assessment may be helpful to direct treatments, and advocate for support and accommodations in school. In the absence of access to formal handwriting assessments, general guidance can be provided to schools to support a child with JIA in the classroom.

### Electronic supplementary material

Below is the link to the electronic supplementary material.

Supplementary Material 1

## Data Availability

The datasets used and/or analysed during the current study are available from the corresponding author on reasonable request.

## Abbreviations

CHAQ: Childhood health assessment questionnaire
DASH: Detailed Assessment of Speed of Handwriting
JIA: Juvenile idiopathic arthritis
OT: Occupational therapist
PT: Physiotherapist
SD: Standard deviation
VAS: Visual analog scale
WPM: Words per minute
