# Supplementary material for: Handwriting speed in juvenile idiopathic arthritis using the detailed assessment of speed of handwriting
Source: Pediatr Rheumatol Online J. 2024 Aug 15;22:75. doi: 10.1186/s12969-024-01013-y (PMC11325831; doi:10.1186/s12969-024-01013-y)
Supplement: Supplementary file 1 — Supplementary Material 1 [file 12969_2024_1013_MOESM1_ESM.docx]

Kristin Houghton

4480 Oak Street, Room K4-123

Vancouver, BC

V6H 3V4

604-875-2437

[khoughton@cw.bc.ca](mailto:khoughton@cw.bc.ca)

July 9, 2024

Pediatric Rheumatology

Editors-in-Chief: Angelo Ravelli, Tadej Avčin

Please accept our revised manuscript entitled “Handwriting speed in juvenile idiopathic arthritis using the Detailed Assessment of Speed of Handwriting”. We have addressed the reviewer comments and believe the suggested changes have enhanced our paper. It has been formatted as per the Short Report guidelines. I confirm that everyone named as an author meets the Uniform Requirements for Manuscripts Submitted to Biomedical Journals criteria for authorship. In compliance with the Helsinki Declaration, the study was approved by the research ethics board at the participating site. I attest to the integrity of the data and agree to pay the applicable publication charges if our manuscript is accepted for publication.

Thank you for receiving our revised manuscript and considering it for review. We appreciate your time and look forward to your response.

Sincerely,

Kristin Houghton MD MSc FRCPC DRCPSC (SEM) Dip Sports Med
